# Unraveling the genome of *Bacillus velezensis* MEP_2_18, a strain producing fengycin homologs with broad antibacterial activity: comprehensive comparative genome analysis

**DOI:** 10.1038/s41598-023-49194-y

**Published:** 2023-12-13

**Authors:** Daniela Medeot, Analía Sannazzaro, María Julia Estrella, Gonzalo Torres Tejerizo, Bruno Contreras-Moreira, Mariano Pistorio, Edgardo Jofré

**Affiliations:** 1 Instituto de Biotecnología Ambiental y Salud (INBIAS), CCT-CONICET-Córdoba, Universidad Nacional de Río Cuarto, 5800 Córdoba, Argentina; 2 Instituto Tecnológico de Chascomús (INTECH), Consejo Nacional de Investigaciones Científicas y Técnicas (CONICET), Universidad Nacional de San Martín (UNSAM), 7130 Chascomús, Argentina; 3 Departamento de Ciencias Biológicas, Facultad de Ciencias Exactas, IBBM (Instituto de Biotecnología y Biología Molecular), CCT-CONICET-La Plata, Universidad Nacional de La Plata, 1900 La Plata, Argentina; 4 Estación Experimental de Aula Dei-CSIC, 50059 Zaragoza, Spain

**Keywords:** Microbiology, Applied microbiology, Microbial genetics

## Abstract

*Bacillus* sp. MEP_2_18, a soil bacterium with high potential as a source of bioactive molecules, produces mostly C16–C17 fengycin and other cyclic lipopeptides (CLP) when growing under previously optimized culture conditions. This work addressed the elucidation of the genome sequence of MEP_2_18 and its taxonomic classification. The genome comprises 3,944,892 bp, with a total of 3474 coding sequences and a G + C content of 46.59%. Our phylogenetic analysis to determine the taxonomic position demonstrated that the assignment of the MEP_2_18 strain to *Bacillus velezensis* species provides insights into its evolutionary context and potential functional attributes. The in silico genome analysis revealed eleven gene clusters involved in the synthesis of secondary metabolites, including non-ribosomal CLP (fengycins and surfactin), polyketides, terpenes, and bacteriocins. Furthermore, genes encoding phytase, involved in the release of phytic phosphate for plant and animal nutrition, or other enzymes such as cellulase, xylanase, and alpha 1–4 glucanase were detected. In vitro antagonistic assays against *Salmonella typhimurium*, *Acinetobacter baumanii*, *Escherichia coli*, among others, demonstrated a broad spectrum of C16–C17 fengycin produced by MEP_2_18. MEP_2_18 genome sequence analysis expanded our understanding of the diversity and genetic relationships within the *Bacillus* genus and updated the *Bacillus* databases with its unique trait to produce antibacterial fengycins and its potential as a resource of biotechnologically useful enzymes.

## Introduction

The genus *Bacillus* is a highly versatile taxon that includes pathogen and plant-growing promoting bacterial members. Particularly, members grouped into the *Bacillus subtilis* and *Bacillus amyloliquefaciens* possess useful characteristics for the biotechnological industry including the production of extracellular enzymes like cellulases, proteases, lipases, xylanases, phytases, and bioactive compounds such as antibiotics. Accurate species identification within the *Bacillus* genus is challenging due to its high genomic diversity and shared phenotypic traits among closely related species. *Bacillus velezensis* (formerly *Bacillus amyloliquefaciens*) was described as a plant growth-promoting bacterium able to produce indole acetic acid, siderophores, and an enormous diversity of antimicrobial compounds, hence *B. velezensis* might be highlighted in the field of rhizosphere microorganisms research in the future^1^.

*Bacillus* carry out effective biological control of pathogens by a combination of mechanisms, including antibiotic production, host-plant resistance induction, and plant growth promotion^2^. The production of antibiotics is the primary mechanism involved in its biological control effect^3^. The antibiotic activity of *Bacillus* species is partly focused on the production of active peptides. Among these antimicrobial metabolites, cyclic lipopeptides (CLP) are the most studied due to their antibacterial, antifungal, and antiviral activities. For instance, *B. velezensis* FZB42, the most biochemical, genetic, and physiologically characterized strain of this species, produces the CLP iturin, surfactin, and fengycin (bacillomycin pliplastin); polyketides and bacteriocins^4,5^.

The plant growth-promoting rhizobacteria (PGPR) *B. amyloliquefaciens* MEP_2_18 (hereafter referred to as MEP_2_18) was isolated from the endorhizosphere of maize growing in saline soil of the Córdoba province (Argentina) and was identified initially as a member of the *B. subtilis* group on the basis of its phenotypic properties and 16S *rRNA* gene sequence analysis^6,7^. Inoculation with MEP_2_18 significantly increased the growth of maize seedlings under normal and saline conditions. Moreover, the cell-free supernatant of MEP_2_18 suppressed, in vitro, the growth of *Fusarium* spp. and *Sclerotinia* spp.^6^ The antifungal compounds produced by MEP_2_18 were characterized and the main antifungal activity was attributed to the production of the CLP iturin A C15^7^.

Previously, we demonstrated that changes in carbon (C) and nitrogen (N) sources and C–N ratios in the culture medium affected quali- and quantitatively the production of CLP in MEP_2_18. Thus, MMOLP medium improved the production of specific antibacterial fengycin^8^. Recently, we have also described in detail the strong antibacterial activity of the C16–C17 fengycin A and B isoforms produced by MEP_2_18 against *Xanthomonas axonopodis* pv*. vesicatoria* (Xav), the phytopathogenic bacterium causing the detrimental bacterial spot disease on tomato plants (*Solanum lycopersicum* L.), and *P. aeruginosa* PA01, an opportunistic pathogen of growing clinical relevance^9^. Images obtained by atomic force microscopy showed prominent alterations in the bacterial surface topography after treatment with fengycins produced by MEP_2_18. Cell damage was evidenced by a decrease in bacterial cell heights and the loss of intracellular content. Furthermore, the viability of MRC-5 human normal lung fibroblasts was not affected by the treatment with the highest concentration of fengycins assayed^9^.

Understanding the taxonomic position of *Bacillus* strains is crucial for unraveling their functional characteristics and evolutionary relationships. Therefore, determining the taxonomic position of MEP_2_18 within the *Bacillus* genus could be essential to gain insights into its evolutionary relationship with closely related strains.

The coordinated actions of FenA and FenD contribute to the production of fengycins, which exhibit potent antimicrobial activity against a range of bacterial pathogens. FenA and FenD play indispensable roles in fengycin biosynthesis. FenA is responsible for the correct assembly of the fengycin peptide chain, while FenD adds the fatty acid moiety, resulting in the formation of the bioactive lipopeptide structure. The genomic organization of the fengycin biosynthetic gene cluster (BGC) in MEP_2_18 could provide valuable insights into the evolution and functional diversity of this important secondary metabolite.

The aim of this work was to analyze the sequence of the complete genome obtained from MEP_2_18, to perform an in silico study comparing genes that are related to antibiosis mechanisms and other peculiarities, with those obtained from databases of the *Bacillus* genus and to establish their taxonomic position by phylogenomic analysis.

The MEP_2_18 genome sequence will allow the reveal of genes encoding new bioactive metabolites with potential biotechnological applications, highlighting novel antimicrobial peptides.

## Results

### Whole genome sequencing and analysis

To understand the mechanisms underlying the biological control capability of bacterial pathogens, the complete genome of MEP_2_18 was sequenced, assembled, and deposited in the GenBank database under the accession number CP042864.2.

**Figure 1:**
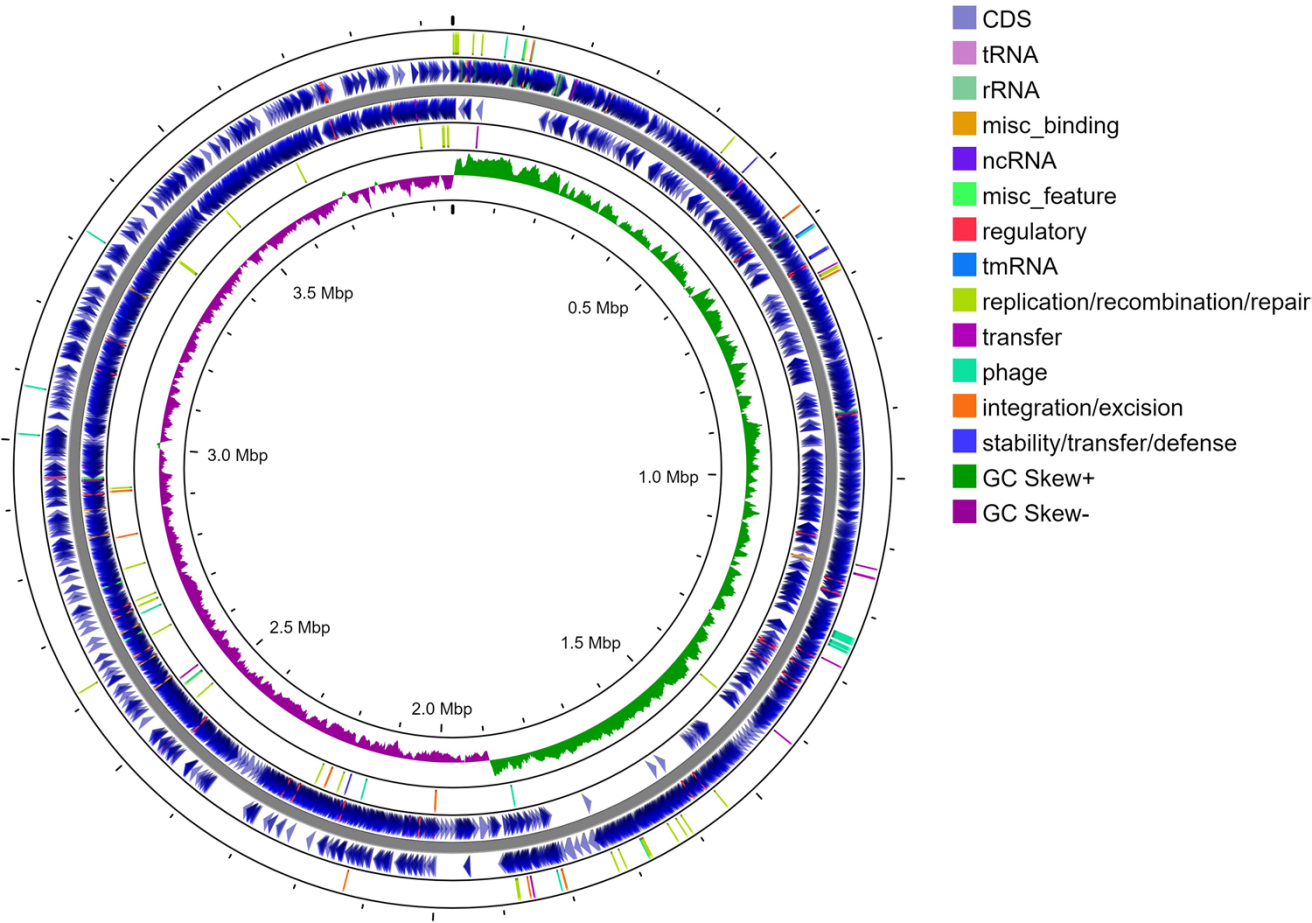
Circular chromosome map of the complete genome of MEP_2_18 generated through Proksee tool (https://proksee.ca), including whole genome annotation and bacterial mobile genetic elements (MGEs) from mobileOG-db^10^.

The genome of MEP_2_18 consists of a single circular chromosome of 3,944,892 bp with a G + C content of 46.59%. In the MEP_2_18 genome, the predicted number of genes was 3,930 and the predicted number of protein-coding sequences (CDS) was 3,474, which is comparable to the 3782 protein-coding reported for the reference genome *B. velezensis* JS25R (CP009679.1), while the number of predicted RNAs was 133 (86 tRNAs and 27 rRNAs). The BlastP analysis targeting plasmid replication and segregation proteins did not yield any significant matches in the MEP_2_18 genome, suggesting the absence of plasmids in this strain. A circular chromosome map of the complete genome of MEP_2_18 was generated through the Proksee tool (Fig. 1) and includes an external ring with the bacterial mobile genetic elements (MGEs) predicted by mobileOG-db^10^.

### Functional annotation

**Figure 2:**
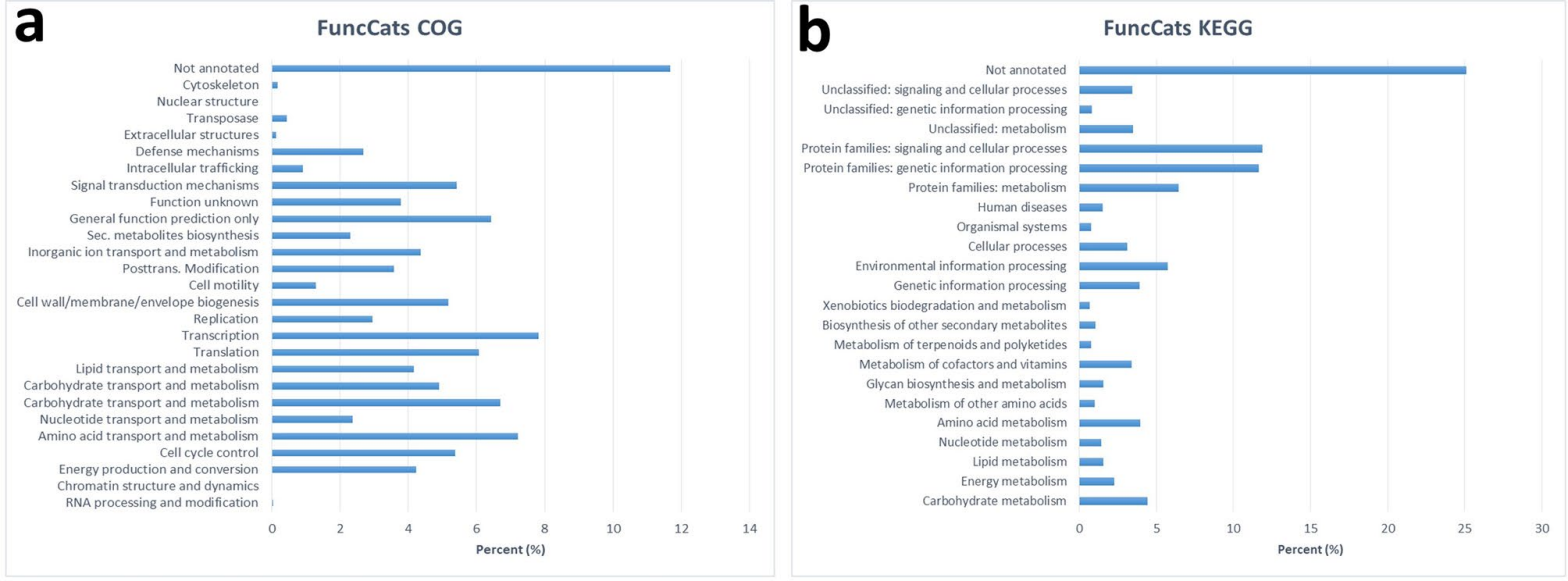
Classification of the cluster of orthologous (**a**) (COG) and (**b**) KEGG functional annotation of MEP_2_18 genome. Different bars indicate the percent (%) of genes assigned to each COG and KEGG functional category.

The functional classification of predicted genes in the MEP_2_18 genome was performed via the EDGAR pipeline. The distribution within the COG categories is shown in Fig. 2a. A total of 26 functional annotations was recorded, with the highest number of genes (426) associated with carbohydrate transport and metabolism, followed by transcription (287), amino acid transport and metabolism (265), translation (223), and signal transduction mechanisms (199). The 84 genes associated with secondary metabolite biosynthesis are highlighted, while 429 genes remain to be annotated. KEGG^11–13^ analysis identified genes belonging to several metabolic pathways (Fig. 2b). The highest number, 528, was recorded for protein families related to signaling and cellular processes, followed by 519 for protein families involved in genetic information processing, and 287 for protein families involved in metabolism. Likewise, 35 were associated with the metabolism of terpenoids and polyketides, and 46 with the biosynthesis of other secondary metabolites. (For complete COG and KEGG^11–13^ functional categories see Supplementary Table 1).

### Phylogenetic analysis

The taxonomic position of the MEP_2_18 strain was initially inferred as *B. amyloliquefaciens* based on a comparison of 16S *rRNA* and *recA* gene sequences with those of known *Bacillus* species available in the GenBank Database^7^. However, to further confirm its taxonomic position, in silico DNA-DNA hybridization (*is*DDH) and average nucleotide identity (ANI) calculations were performed after the complete genome sequencing.

**Figure 3:**
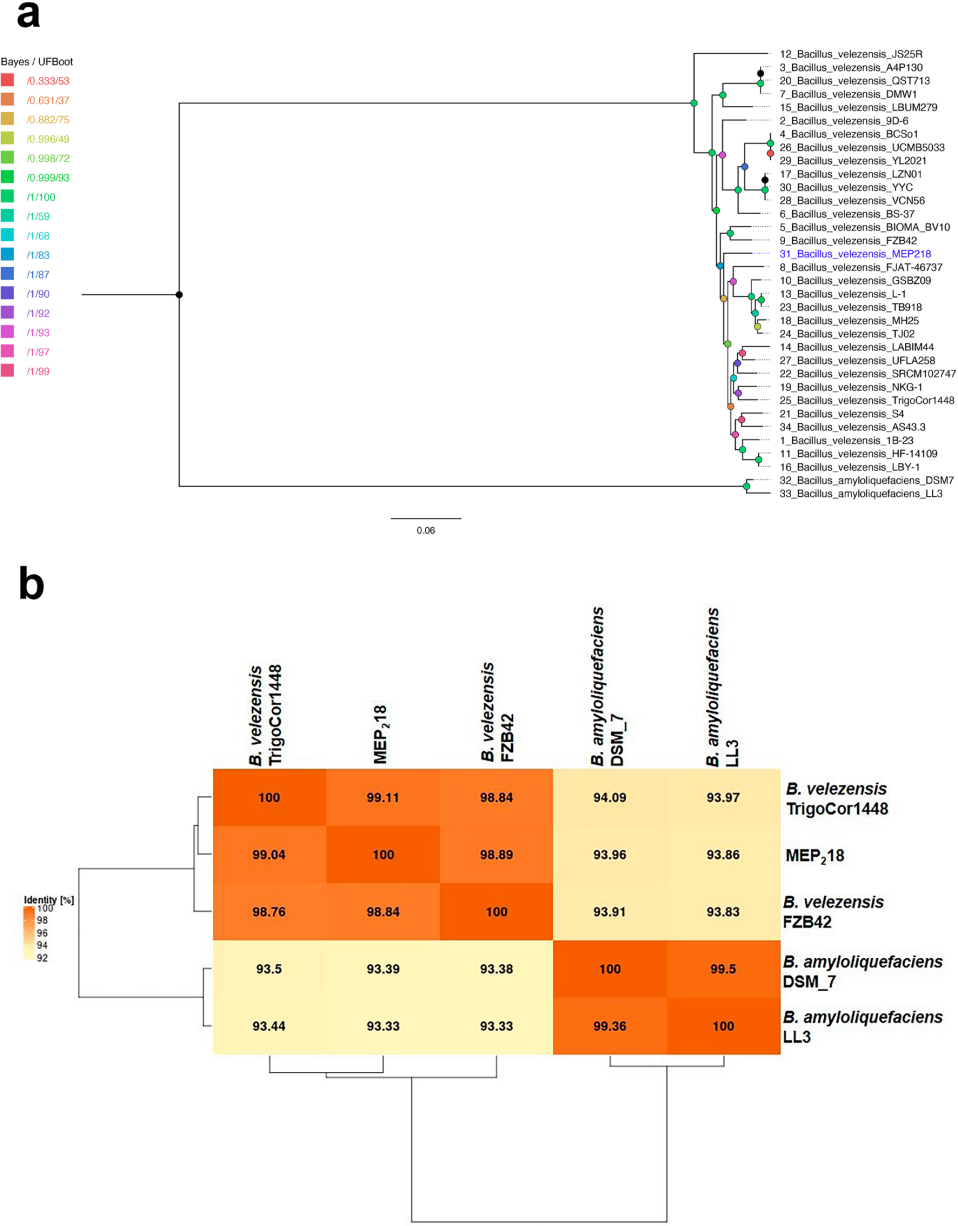
(**a**) Maximum likelihood phylogenetic tree of 52 concatenated CDS aligned sequences shared by 32 *Bacillus velezensis* and 2 *Bacillus amyloliquefaciens* strains. Bayes branch support and UFBoot2 bootstrap values are indicated by node color. The MEP_2_18 strain is shown in blue. The tree was midpoint-rooted. (**b**) Average nucleotide identity (ANI) mean heatmap generated using the EDGAR tool depicts the genomic relatedness resulting from a multi-genome comparison among strains of *Bacillus velezensis* and *Bacillus amyloliquefaciens*.

The *is*DDH analysis, conducted using the Type (Strain) Genome Server^14^ revealed that the clustering yielded 10 species clusters, and the MEP_2_18 strain was assigned to *Bacillus velezensis*. These results indicate a close genomic relationship between MEP_2_18 and other *Bacillus velezensis* strains, confirming its taxonomic affiliation within this species rather than with *Bacillus amyloliquefaciens* (See Supplementary Table 2). Subsequently, and to precise the actual phylogenetic position of MEP_2_18 among the *B. amyloliquefaciens* and *B. velezensis* species, a phylogenetic tree was constructed using selected high-quality markers identified by GET_PHYLOMARKERS^15^, specifically concatenated nucleotide sequences from 52 CDS. During this process, we observed that *B. amyloliquefaciens* GKT04 clusters with *B. velezensis* strains in the trees, suggesting a possible classification error. The phylogenetic tree provided robust support for the inclusion of MEP_2_18 within the *Bacillus velezensis* species. High Bayes support and bootstrap values underscored the reliability of the clustering, reaffirming the accurate placement of MEP_2_18 within the identified species (Fig. 3a). Furthermore, the EDGAR pipeline yielded validated ANI values for MEP_2_18, and the comparison with related *B. velezensis* genomes revealed ANI percentages above the 98% threshold. Taking into account that the recommended cut-off point of 70% *is*DDH for species delineation corresponded to 95% ANI^16^, these results confirm the MEP_2_18 strain's significant genetic similarity with other *B. velezensis* strains, including JS25R, FZB42 and TrigoCor1448 (Fig. 3b).

### Comparative genome analysis

**Figure 4:**
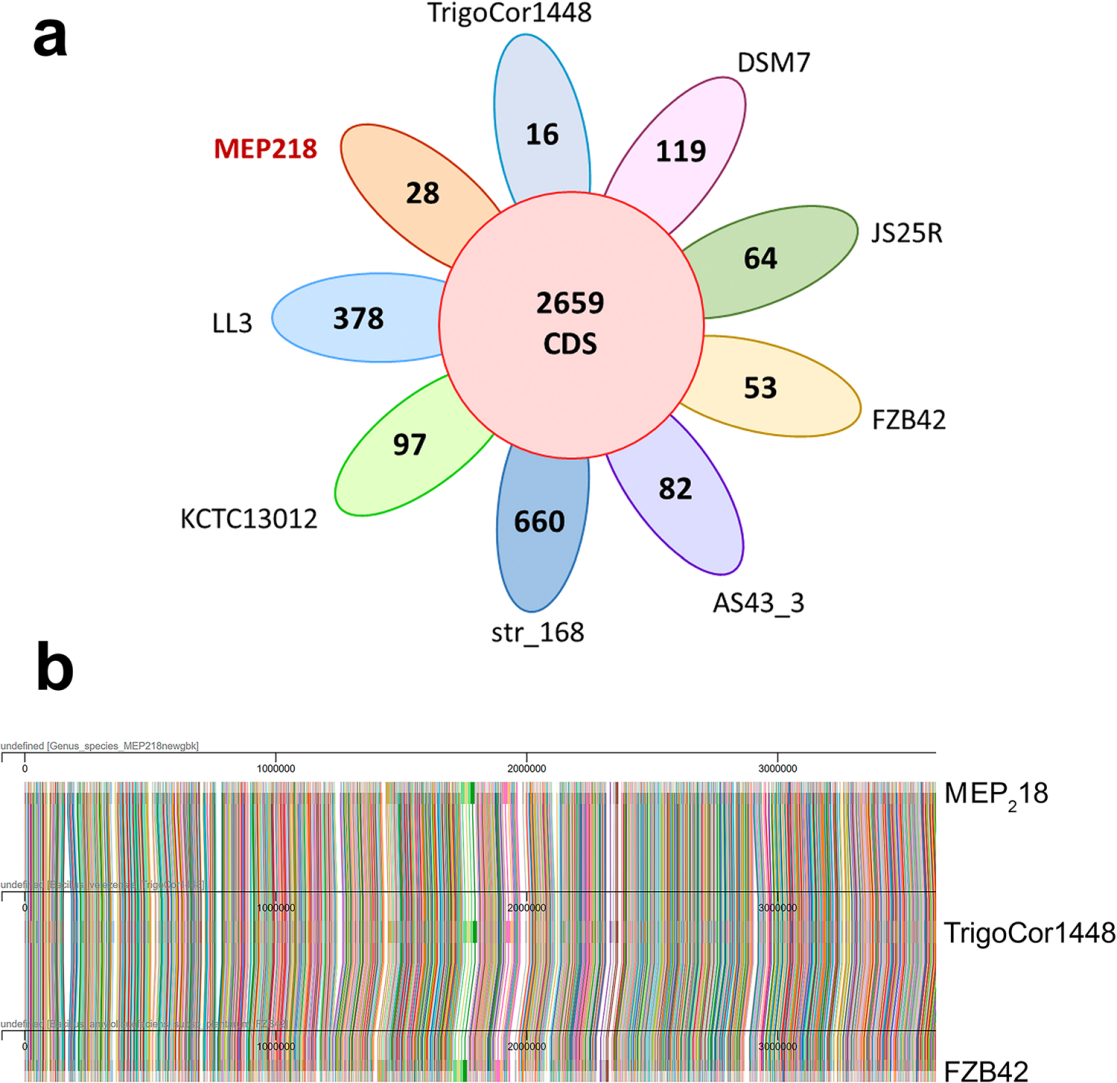
(**a**) Pan-genome flower. The number of the core CDS is shown in the central circle while, each petal shows the number of specific CDS (singletons) of each *Bacillus* strain. (**b**) Mauve plot showing a global macro-synteny overview, allowing to detect collinearity and the conservation of gene order between the genomes of the *B. velezensis* strains FZB42, TrigoCor1448 and MEP_2_18. Each cluster of the core-genome is materialized as a link between genomes.

When MEP_2_18 was set as the reference genome in the EDGAR software, the core genome was found to consist of 2659 CDS, while the pan-genome comprised 6291 CDS. Figure 4a shows 28 singletons specific to *B. velezensis* MEP_2_18 (CP042864) in comparison with *B. amyloliquefaciens* DSM7 (FN597644), *B. amyloliquefaciens* LL3 (CP002634), *B. subtilis* subsp *subtilis* str 168 (AL009126), *B. velezensis* AS43 3 (CP003838), *B. velezensis* FZB42 (CP000560.2), *B. velezensis* TrigoCor1448 (CP007244.1), *B. velezensis* KCTC13012NZ (LHCC01000009), and the reference strain *B. velezensis* JS25R (CP009679). Upon comparing the singleton numbers, it was evident that MEP_2_18, TrigoCor1448, and FZB42 were the most closely related strains, as they shared the highest number of specific singletons.

To further compare in-depth the genomes of the closely related strains MEP_2_18, TrigoCor1448, and FZB42, we conducted an independent Mauve plot analysis using the PanExplorer pipeline^17^. The Mauve plot (Fig. 4b) illustrated the physical positions of core genes within the three selected genomes, enabling the evaluation of synteny or gene order conservation between these genomes.

The absence of an abrupt color change in the Mauve plot when comparing the genome of MEP_2_18 with those of the FZB42 and TrigoCor1448 strains indicates collinearity in the gene order between MEP_2_18 and the other two strains. Additionally, MEP_2_18 has unique genes that are not present in FZB42 and TrigoCor1448, leading to changes in the gene clusters. Conversely, there might be some core genes present in FZB42 and TrigoCor1448 that are missing in MEP_2_18, causing a difference in the gene order between the strains. Over time, genetic differences can accumulate between different strains of the same species, leading to changes in gene order. Thus, MEP_2_18 might have followed the same evolutionary path compared to FZB42 and TrigoCor1448, resulting in the observed collinearity in the Mauve plot.

### Presence of integrative and conjugative elements (ICEs), integrative and mobilizable elements (IMEs), genomic islands, and antimicrobial resistance (AMR) annotations

No ICEs/IMEs were predicted in the MEP_2_18 genome with the web-based tool ICEfinder^18^. This result could be due to a limitation of the method or to the absence of ICEs/IMEs in the genome.

Nevertheless, by using mobileOG-db tool from Proksee^19^ system (https://proksee.ca/), 105 mobile genetic elements (MGEs) were detected throughout the entire genome and classified as: 16 elements for integration/excision, 43 elements for replication/recombination/repair, 30 elements for phage, 6 elements for stability/transfer/defense, and 10 elements for transfer (Fig. 1).

**Figure 5:**
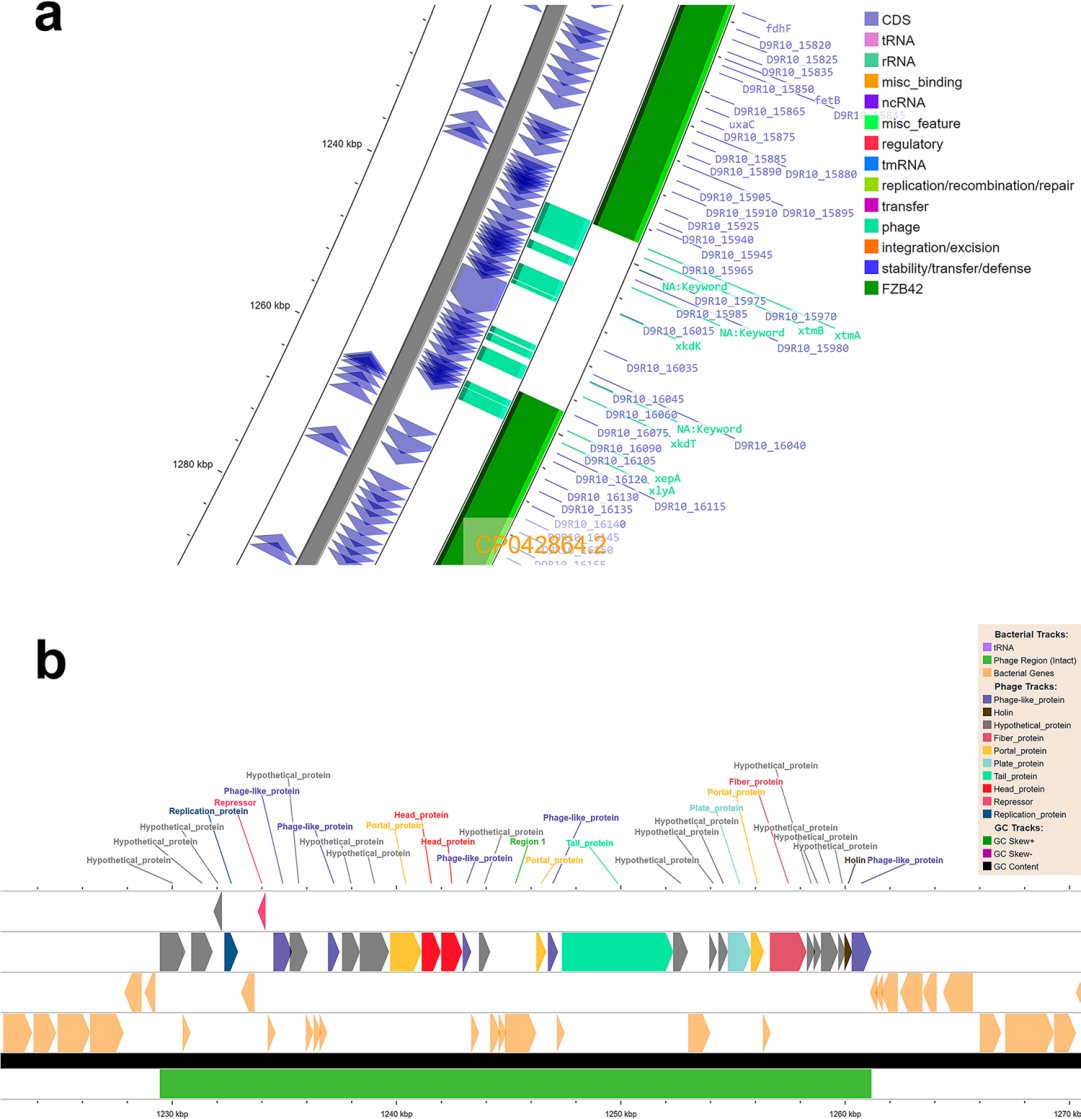
(**a**) Zoomed genomic region of the MEP_2_18 chromosome between 1220 and 1280 kbp showing phage elements present in MEP_2_18 and absent in *B. velezensis* FZB42. (**b**) Map of *Brevibacillus* phage Osiris region detected in MEP_2_18 chromosome by using PHASTEST.

A genomic region of 31.7 Kb (from 1,229,447 to 1,261,166 bp) containing the intact elements of the *Brevibacillus* phage Osiris was detected by using the mobileOG-db and the PHASTEST^20^ web server (Fig. 5a). The phage elements comprise 30 CDS encoding replication, repressor, phage-like, portal, head, tail, plate, fiber, holin, and hypothetical proteins. Furthermore, the sequence of the uncharacterized protein D9R10_16075 from MEP_2_18 produced significant alignments with pyocin knob domain-containing proteins from *B. velezensis*. When BLAST analysis was performed, this element was absent in the reference strain FZB42 (Fig. 5b). In addition to the elements from *Brevibacillus* phage Osiris, DBSCAN-SWA^21^ detected four regions containing phage elements from *Planktothrix* phage (from 1,139,751 to 1,172,932 bp), *Bacillus* phage Grass (from 1,813,877 to 1,820,091 bp), *Bacillus* phage SPBc2, (from 2,123,672 to 2,132,962 bp), and *Staphylococcus* phage (from 2,268,994 to 2,275,248 bp). The T7SS effector LXG polymorphic toxin (D9R10_00105), a component of the TSS7 secretion system, was detected among the phage elements of *Bacillus* phage SPBc2.

The MEP_2_18 genome possesses six genomic islands predicted by IslandViewer 4 tool^22^. These islands are located in different zones across the genome with lengths ranging from 4,282 to 18,842 bp and contain some genes of potential biotechnological interest like 3-phytase (See Supplementary Table 3). In contrast, six prophage elements, and five IScluster/Tn were detected in the MEP_2_18 genome when the VRprofile2^23^ tool was used (https://tool2-mml.sjtu.edu.cn/VRprofile/VRprofile.php) (See Supplementary Table 4). During the search for antibiotic resistance-associated mobilome, both the VRprofile2 and CARD tools detected the presence of the *clbA* antibiotic resistance gene (ARG). This gene is associated with the drug classes of Oxazolidinone, Phenicol, Lincosamide, Streptogramin A, and Pleuromutilin. The *clbA* gene was located in the genomic positions between 539,152 and 540,201 bp in the MEP_2_18 genome. CARD detected another six ARG, but the percentage of identity of the matching region was less than 63% (See Supplementary Table 5).

### Commercially useful enzymes and secondary metabolites clusters

MEP_2_18 genome was analysed to find novel homologs for known Commercially Useful Enzymes (CUEs) in the MetaBioME^24^ web resource. This analysis detected numerous homolog genes encoding CUEs. Among these, phytases are prominent for their role in phytic acid phosphorus mobilization, cellulases for cellulose hydrolysis, β-1-4 xylanases for xylan hydrolysis, β-mannanases for mannan, glucomannan, and galactomannan hydrolysis, and α-amylases for starch hydrolysis.

Gene clusters related to the secondary metabolite synthesis in the MEP_2_18 genome were identified using three web server tools: antiSMASH 7.0^25^, BAGEL4^26^ and PRISM4^27^. Among the eleven putative gene cluster regions found with antiSMASH, four encoded for NRPS (non-ribosomal peptide synthetase), three encoded for transAT-PKS (trans-acyl transferase polyketide synthetase), two encoded for terpene, one encoded for PKS-like (Type III polyketide-like synthetase), two encoded for T3PKS (Type III polyketide synthetase), and one encoded the ladderane plantazolicin; considering that the same genomic region could contain more than one type of secondary metabolite clusters predicted by antiSMASH (Table 1). Among the four NRPS clusters, the similarity with reported gene clusters for bacillibactin, fengycin, surfactin, and bacillaene were 100, 100, 91, and 100%, respectively. In addition, among the three transAT-PKS clusters, the similarity with reported gene clusters for difficidin, macrolactin H, and bacillaene, were 73, 100, and 100%, respectively (Table 1).

**Table 1:** Secondary metabolite gene clusters detected in the *Bacillus velenzesis* MEP_2_18 genome using anti-SMASH, BAGEL 4, and PRISM4 tools.

Region	Type	From (bp)	To (bp)	Most similar known cluster	Similarity (%)	Web Server Tool
1	Class II/III confident bacteriocin	299,913	307,655			PRISM4
2	Bacteriocin class II, AMP	296,600	316,735	LCI	93.5	BAGEL4
3	NRPS	318,870	383,727	Surfactin	91	Anti-SMASH
3	NRPS	338,349	375,772	Surfactin		PRISM4
4	Ladderane, LAP	683,329	745,623	Plantazolicin	91	Anti-SMASH
4	LAP, Bacteriocin class I, microcin	720,218	744,670	Plantathiazolicin (Plantazolicin)	100	BAGEL4
5	PKS-like	957,169	998,389	Butirosin A/Butirosin B	7	Anti-SMASH
6	terpene	1,084,021	1,101,191			Anti-SMASH
7	transAT-PKS	1,405,021	1,492,862	Macrolactin H	100	anti-SMASH
7	PKS-like	1,408,620	1,476,696	polyketide		PRISM4
8	transAT-PKS, T3PKS, transAT-PKS-like, NRPS	1,712,368	1,814,270	Bacillaene	100	Anti-SMASH
8	polyketide, nonribosomal peptide	1,731,486	1,801,597	Bacillaene		PRISM4
9	NRPS, transAT-PKS, betalactona	1,881,370	2,015,609	Fengycin	100	Anti-SMASH
9	polyketide, nonribosomal peptide	1,900,758	1,938,007	Fengycin		PRISM4
9	nonribosomal peptide	1,955,815	2,003,009	Fengycin		PRISM4
10	terpene	2,045,361	2,067,244			Anti-SMASH
11	T3PKS	2,141,042	2,182,142			anti-SMASH
12	transAT-PKS-like, transAT-PKS	2,319,388	2,405,455	Difficidin	100	Anti-SMASH
12	polyketide	2,315,527	2,390,037			PRISM4
13	Bacteriocin class I, Quorum sensing peptide pheromone	3,010,904	3,031,075	ComX1	100	BAGEL4
13		3,020,900	3,021,967	ComX/ComQ		PRISM4
14	NRPS, bacteriocin	3,025,035	3,076,823	Bacillibactin	100	Anti-SMASH
14	NRPS	3,044,623	3,054,335			PRISM4
15	Bacteriocin class I, Head-to-tail cyclized peptides	3,061,079	3,081,271	Amylocyclicin	100	BAGEL4
15	Bacterial head-to-tail cyclized peptide	3,068,118	3,071,415			PRISM4
16	Bacilysin	3,608,225	3,622,751			PRISM4

BAGEL4 and PRISM4 found a gene cluster similar to ComX/ComQ required for the production of ComX pheromone related to quorum sensing. While antiSMASH only detected the plantazolicin cluster among other bacteriocins, BAGEL4, a web server utilized for identifying and visualizing gene clusters involved in the biosynthesis of Ribosomally synthesized and Post-translationally modified Peptides (RiPPs) and (unmodified) bacteriocins, along with PRISM4, identified two additional bacteriocin gene clusters: LCI and amylocyclicin. Interestingly, these clusters were absent in the antiSMASH report (Table 1).

The fengycin gene cluster identified in MEP_2_18 exhibited significant similarity to previously characterized fengycin biosynthetic gene clusters in other *Bacillus* species suggesting a conserved genetic architecture and functional importance of the fengycin biosynthetic pathway.

### Peculiarities of the MEP_2_18 fengycin cluster

FenA and FenD are two key proteins involved in the synthesis of fengycin, a CLP with potent antimicrobial properties^28^.

**Figure 6:**
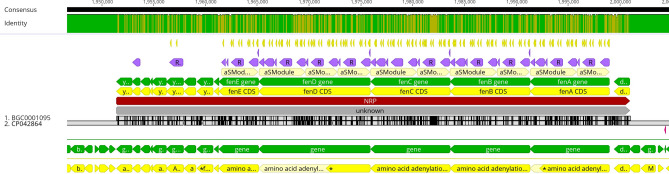
Pairwise alignment of the gene cluster responsible for fengycin synthesis from MEP_2_18 genome (downwards from the 2.CP042864) and the fengycin biosynthetic gene cluster BGC0001095 of *Bacillus velezensis* FZB42 (upwards from the 1.BGC0001095). Alignment was performed with the Geneious Prime^®^ 2023.1.1 sequence analysis software. Gene sequences are represented in green arrows and the sequences of CDS in yellow arrows. Frameshifts are indicated with (*) in the MEP_2_18 CDS sequences of *fenA* and *fenD*.

Using Geneious Prime^®^ 2023.1.1 sequence analysis software, we successfully located the cluster of genes responsible for fengycin synthesis in the MEP_2_18 genome between 1,951,393 and 2,000,936 bp positions. Subsequently, we performed a pairwise alignment of this BGC with the known fengycin BGC0001095 of *B. velezensis* FZB42, revealing a remarkable level of similarity. This alignment exhibited 98.6% sequence identity and 98.6% identical sites between the two gene clusters, indicating a high degree of conservation. However, it is important to note that during our analysis, we observed frameshifts in the CDS of *fenA* and *fenD* within the fengycin BGC of MEP_2_18 relative to that of *B. velezensis* FZB42. Specifically, *fenA* exhibited a single gap, while *fenD* displayed two gaps. These frameshifts suggest the presence of small insertions or deletions in these genes, which might have functional implications for the fengycin biosynthetic pathway (Fig. 6).

### Antimicrobial activity of C16–C17 fengycin produced by *B. velezensis* MEP_2_18 against bacterial pathogens

**Figure 7:**
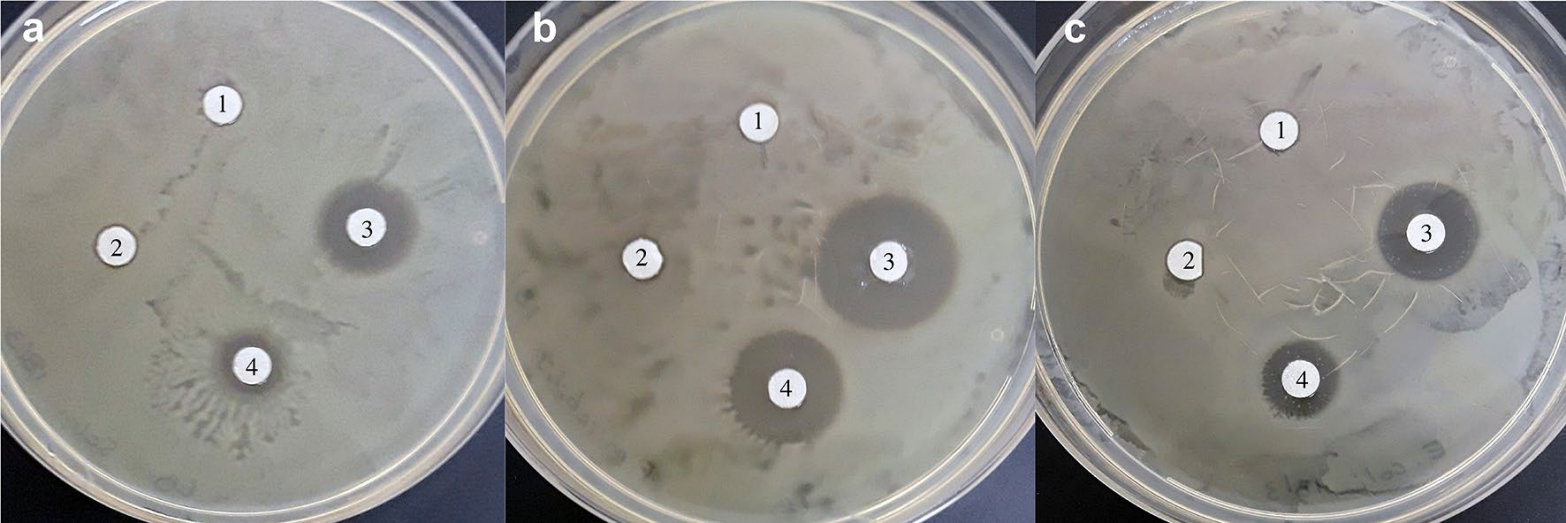
Antibacterial activity of CLP and C16–C17 fengycin produced by MEP_2_18 against *Salmonella typhimurium* ATCC 14028 (**A**), *Proteus mirabilis* (**B**), and *E. coli* (**C**) after 24 h at 37 °C. Paper discs were imbibed with 20 µL of: CLP from JH642 (1), supernatant 20X from MEP_2_18 (2), CLP MEP_2_18 50x (3) and C16–C17 fengycin (stock 10 mg/mL) (4).

In addition to the previously reported antifungal and antibacterial spectra of the CLP produced by MEP_2_18^6–9^, we investigated whether the total fraction of CLP or the purified C16–C17 fengycin produced by MEP_2_18 could also inhibit the growth of human and animal bacterial pathogens. Among the pathogens tested, the CLP produced by MEP_2_18 were active in inhibiting the growth of Gram-negative bacilli, including *Salmonella typhimurium* ATCC 14028, *Proteus mirabilis*, and *Escherichia coli* (Fig. 7). Notably, the inhibitory effects were specifically exerted by the fraction of CLP containing the C16–C17 fengycin.

**Figure 8:**
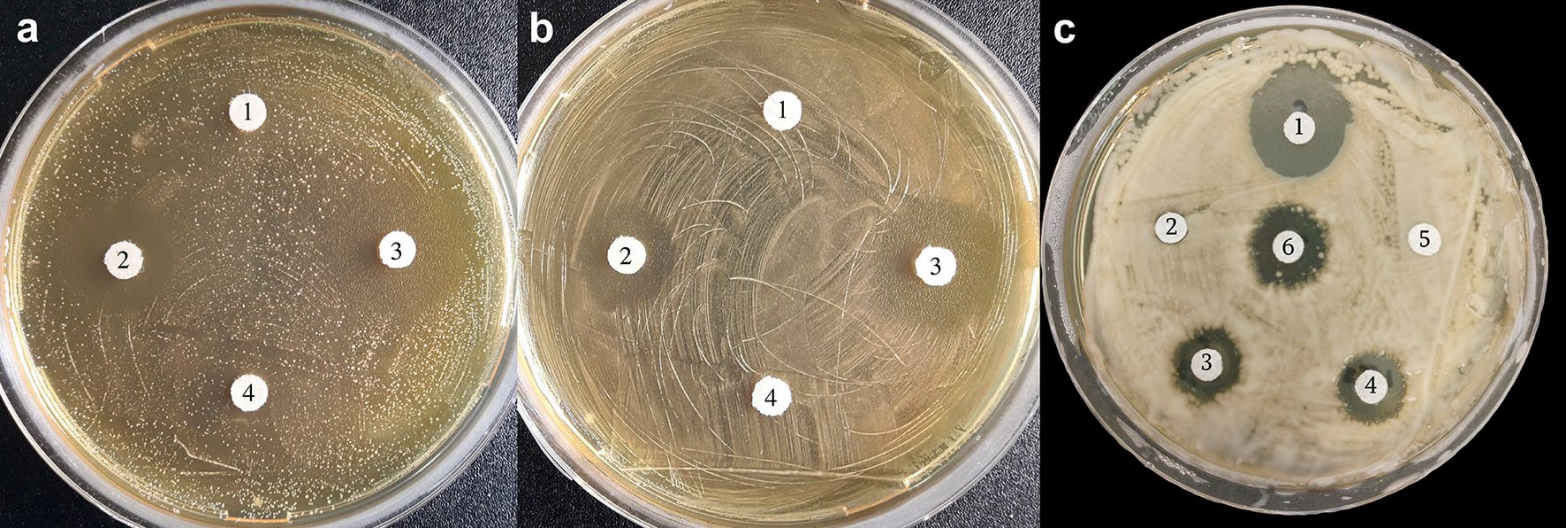
Antibacterial activity of CLP and C16–C17 fengycin produced by MEP_2_18 against *Streptococcus uberis* SU33 (**A**) and SU 56 (**B**). Paper discs were imbibed with 10 µL of: CLP from JH642 (1), C16–C17 fengycin (stock 10 mg/mL) (2), CLP MEP_2_18 50x (3), and methanol 100% v/v (4). *Acinetobacter baumanii* Ab242 (C). Paper discs were imbibed with 10 µL of: Ceftazidime (stock 0.4 mg/mL) (1), fengycins F22-32 (stock 10 mg/mL) (2), C16–C17 fengycin (stock 5 mg/mL) (3), CLP MEP_2_18 (stock 30 mg/mL) (4), fengycins F30 (stock 5 mg/mL) (5) and tobramycin (stock 3 mg/mL) (6).

When we performed antagonism assays against strains of *S. uberis* isolated from bovine mastitis (UB33 and UB56), as well as against *A. baumannii* Ab242—a clinically multidrug-resistant strain— it was observed that, at the concentrations tested, the CLP of MEP_2_18 effectively inhibited bacterial growth, with C16–C17 fengycin being the active fraction (Fig. 8). The remaining fengycins (referred to as F22–32 and F30 in Fig. 8C) exhibited no antibacterial activity. The obtained results were compared with the hospital-used antibiotics Tobramycin and Ceftazidime.

## Discussion

Exploring the genomes of *Bacillus* strains with notable biotechnological and pharmaceutical characteristics presents a valuable opportunity to unravel the molecular mechanisms involved in the biosynthesis of secondary metabolites^29^. Particularly, it allows for the identification of key genes and genetic elements involved in producing specific and useful antimicrobials.

The size of the genome and the G + C content, are consistent with the sizes and G + C contents of other sequenced genomes of *Bacillus* species since the median total length across *B. velezensis* genomes recorded by the NCBI is 3.96079 Mbp, with a median GC% of 46.4%. In this study, we conducted a comprehensive analysis of the MEP_2_18 genome and reassigned its taxonomic position as *Bacillus velezensis*. Additionally, we employed a genomic approach to conduct a detailed gene analysis. The comparative genome analysis enabled us to explore the genetic relatedness between phylogenetically similar species that produce diverse metabolites. This analysis also provided crucial information about relevant traits present in the core genome, which functionally important genetic elements are contributing to the strains' overall biological characteristics. The *is*DDH and ANI results highlighted the close genomic relatedness between MEP_2_18 and *B. velezensis* strains. These findings provide valuable insights into the taxonomic position of MEP_2_18 within the *Bacillus* genus, suggesting a close evolutionary relationship to other *B. velezensis* and *B. amyloliquefaciens* strains. GET_HOMOLOGUES^30^ and their package GET_PHYLOMARKERS^15^, offer powerful tools for identifying and analyzing phylogenetic markers, resolving taxonomic uncertainties and improving species classification. GET_PHYLOMARKERS selects appropriate genomic regions or genes that can serve as robust phylogenetic markers for a specific group of organisms, such as bacteria. These markers are typically highly conserved and evolutionarily informative, making them reliable indicators of evolutionary history. In this study, we successfully reclassified the *B. amyloliquefaciens* MEP_2_18 strain within the *B. velezensis* species using TYGS and PHYLOMARKERS-based phylogenetic analysis. This finding enhances our understanding of the genetic diversity and evolutionary relationships within the *Bacillus* genus.

We found several mobile genetic elements in MEP_2_18 genome. ICEs and IMEs are important bacterial mobile genetic elements and are integrative to the bacterial chromosome. ICEs and IMEs encode a conjugation machinery and genes for antibiotic resistance and virulence factors. The ICE can confer the host with selective advantages for bacterial adaptation and evolution and are self-transmissible between bacterial cells. Intact elements of the *Brevibacillus* phage Osiris^31^ were detected within the genome of MEP_2_18. Additionally, some phage elements, including the T7SS effector LXG polymorphic toxin, were also evidenced in the MEP_2_18 genome. The LXG toxin, a component of the TSS7b secretion system found in Firmicutes, has been reported to play a key role during plant root colonization by beneficial *B. velezensis* SQR9. YukE (the homolog protein of LXG) upon inserting into root plasma membrane causes root iron leakage promoting root colonization by *B. velezensis* SQR9^32^.

*B. velezensis* is known for its ability to produce a diverse array of enzymes with biotechnological applications and bioactive secondary metabolites, including fengycins, non-ribosomal peptides, and polyketides^33^. These metabolites exhibit significant antimicrobial properties and have attracted attention for their potential applications in various fields^34^. *B. velezensis* MEP_2_18 displays a remarkable antibacterial potential, as its genome harbors multiple coding sequences responsible for the biosynthesis of polyketide and non-ribosomal peptide antibiotics. However, identifying all potential gene clusters for secondary metabolites in newly sequenced genomes has posed significant challenges, mainly due to the complex biochemical nature, the involvement of unknown enzymes, and the dispersed availability of specialized bioinformatics tools and resources needed for such analyses^29^.

In the MEP_2_18 genome, the 2.29% of the genes was associated with the functional category COG of secondary metabolite biosynthesis. This percentage is similar to the 2.39% found in FZB42 strain. Using three different web server tools, we found 16 regions in the genome of MEP_2_18 dedicated to the synthesis of secondary metabolites. Similarly, the genome of FZB42 revealed the presence of 13 gene clusters engaged in the non-ribosomal and ribosomal synthesis of secondary metabolites with potential antimicrobial properties. These clusters collectively account for approximately 10% of the entire genome^4^. The detection of fengycin gene cluster in MEP_2_18 highlights the strain's potential to synthesize fengycins with strong antimicrobial properties. Fengycins produced by MEP_2_18 have demonstrated efficacy against several bacterial pathogens, making them promising candidates for the development of novel antimicrobial agents^9^. The presence of NRPS and PKS gene clusters in MEP_2_18 further underscores the biosynthetic potential of this strain. NRPSs and PKSs are involved in the production of structurally diverse bioactive compounds, including antimicrobial peptides and polyketides with pharmaceutical and agricultural applications. The identification of these biosynthetic gene clusters in MEP_2_18 suggests a rich repertoire of secondary metabolite production, which could contribute to the strain's ecological fitness and survival in various environments.

The high level of sequence conservation observed between the fengycin BGC of MEP_2_18 and the BGC0001095 cluster of *B. velezensis* FZB42 highlights the evolutionary stability and importance of this gene cluster across different strains. The conservation of key genes involved in fengycin synthesis suggests that the production of this antimicrobial compound is a conserved trait in *B. velezensis*. However, the frameshifts observed in *fenA* and *fenD* CDS of MEP_2_18 are an intriguing finding. Such frameshifts can disrupt the reading frame and potentially alter the functionality of the encoded proteins, which could have significant consequences for the biochemical pathways and the resulting metabolites.

FenA and FenD play critical roles in different steps of the fengycin biosynthetic pathway, contributing to the production of this bioactive compound. FenA is an essential enzyme responsible for the assembly and activation of the fengycin peptide chain. It belongs to the family of NRPS, which are large multi-domain proteins involved in the biosynthesis of various bioactive peptides and secondary metabolites. The primary function of FenA is to catalyze the stepwise addition of amino acids to the growing fengycin peptide chain. It recognizes and activates individual amino acids, ensuring their incorporation into the correct positions of the peptide sequence. FenA consists of several functional domains, including adenylation, thiolation, and condensation domains^28^.

FenD is another important protein involved in fengycin synthesis which catalyzes the acylation of the fengycin peptide chain with a fatty acid moiety, resulting in the formation of the lipopeptide structure characteristic of fengycins. FenD contains an acyltransferase domain responsible for the transfer of a fatty acid onto the thiolation domain of the growing fengycin peptide chain. This acylation step is crucial for the final structure and activity of fengycin, as it imparts the amphiphilic nature to the lipopeptide, allowing it to interact with and disrupt microbial membranes^35^. The fatty acid incorporated by FenD can vary depending on the specific fengycin variant produced by the bacterial strain. The type and length of the fatty acid can influence the physicochemical properties and antimicrobial activity of fengycins, contributing to their broad-spectrum effectiveness against various pathogens^33^.

The fengycin biosynthetic gene clusters (BGCs) were categorized into four groups (A, B, C and Others)^36^, wherein group A contained all the biosynthetic genes (*fenA* to *fenE*). However, BGCs in groups B, C, and Others were found to be incomplete, lacking up to three of the biosynthetic genes. Interestingly, the strains carrying these incomplete fengycin BGCs showed close phylogenetic relationships. This suggests that the deletions in the BGCs were conserved within a specific clade, thus presumably preceding the divergence^36^. In the fengycin operon, large or complete deletions are frequent in other *Bacillus* strains^37^. In this context, it is plausible to speculate that the frameshifts detected in *fenA* and *fenD* from MEP_2_18 may contribute to the unique and exclusive antibacterial property of fengycin C16–C17. The resulting alterations in protein structure and function could potentially lead to the production of fengycin variants with distinct antimicrobial properties, including enhanced activity against a broader range of bacterial pathogens. Several phylogenetically related *Bacillus* strains harboring group B fengycin BGCs showed a frameshift at positions 3126–3127 of the *fenD* gene, indicating a potential evolutionary characteristic of this clade. Consequently, these frameshift mutations lead to the translation of an alternative protein sequence that lacks the functional domains of FenD^36^.

Furthermore, in recent studies we reported that fengycins produced by MEP_2_18 exhibit a unique antibacterial action against a broad spectrum of bacterial pathogens^9^. Interestingly, this observation raises the question of whether the frameshifts observed in *fenA* and *fenD* could be linked to the distinctive antibacterial properties exhibited by fengycins from MEP_2_18. Further functional studies involving genetic manipulation of *fenA* and *fenD* in *Bacillus* strains could provide direct evidence of the causal relationship between frameshifts and the antibacterial activity of fengycins.

Future investigations focusing on the characterization of the fengycin cluster from MEP_2_18, as well as the NRPS and PKS gene clusters, are warranted to understand the biosynthesis, regulation, and structural diversity of these metabolites. Functional studies involving gene knockouts, complementation assays, and heterologous expression could provide valuable insights into the roles of these biosynthetic pathways and the potential applications of the synthesized compounds. Additionally, further research is warranted to explore the functional potential and ecological significance of the MEP_2_18 strain within the *B. velezensis* species.

In summary, the antibacterial effect exherted by C16–C17 fengycins produced by *B. velezensis* MEP_2_18, along with the frameshifts observed in *fenA* and *fenD* CDS, suggests a potential relationship between these genetic variations and the exclusive antimicrobial properties of C16–C17 fengycins. Further functional studies are required to determine the impact of gene sequence gaps on fengycin biosynthesis and to unravel the precise mechanisms underlying this phenomenon. Understanding the interplay between genetic variations and antimicrobial activity in fengycin-producing *Bacillus* strains could provide valuable insights for the development of novel antimicrobial agents targeting a wide range of bacterial pathogens.

## Methods

### Strains used in this study

MEP_2_18, a strain with biocontrol properties against bacterial and fungal phytopathogens, was isolated from a saline soil of the south of the Córdoba province, Argentina^6^. The genomic sequences of *Bacillus* strains, used for performing comparative studies, were downloaded from the National Center for Biotechnology Information (NCBI) database.

*Acinetobacter baumanii* Ab242^38^, *Streptococcus uberis* strains^39^ UB33 and UB56, *Salmonella typhimurium* ATCC 14028, *Proteus mirabilis* and *Escherichia coli* were grown in LB or in Muller-Hinton agar for direct antagonism assays.

*P. mirabilis* and *E. coli* strains were previously isolated by our laboratory from cecal of broiler chickens and identified by whole-cell matrix-assisted laser-desorption-ionization–time-of-flight mass spectrometry (MALDI-TOF MS) as described in López et al.^40^.

*Bacillus subtilis* JH642 (with deletions in *trpC2* and *pheA1*), a derivative non-lipopeptide producer strain (genotypically *sfp0*), was used as a negative control^41^.

### Genome sequencing, assembly and annotation

High Molecular weight DNA was extracted from an overnight culture of MEP_2_18, grown at 30 °C and 150 rpm in LB^42^, using the Qiagen Genomic tip kit (Qiagen Part Numbers: 10243, 19060). American Bio Lysozyme 100 mg/µL (Part number: ABO1178-00005) was used according to Qiagen’s protocol to lyse the bacterial cell wall. DNA was visualized on a 0.75% Blue Pippin pulse field gel and qubited (Thermofisher PN: Q32854) for accurate concentration. Samples were sheared using Covaris G-tube (PN:520079) to 20 kb following their recommended protocol. DNA samples were visualized using a Bioanalyzer 12,000 DNA kit to ensure accurate shearing to 20 kb. Samples were prepped using 10 µg of DNA following Pacific Biosciences 20 kb Smartbell protocol using Pacific Biosciences library prep kit (PacificBiosciencesPN:100-259-100). Samples were sequenced on the Pacific Biosciences RSII. The contig N50 was 3.9 Mb, and the genome coverage was 385.5X. The whole-genome sequence was assembled using Canu 1.8 with default parameters^43^.

ORFs functional annotation was carried out through the utilization of three different servers: GenDB^44^, RAST (Rapid Annotation using Subsystem Technology)^45^, and PGAP (NCBI Prokaryotic Genome Annotation Pipeline). These servers are well-known for their capability to provide comprehensive and accurate functional annotations for genomic sequences in prokaryotic organisms. The data was deposited with the followed ID: BioSample SAMN10231745 and BioProject PRJNA495811.

### Phylogenomics analysis

In silico DNA-DNA hybridyzation^46^ was performed at the GGDC server (https://ggdc.dsmz.de/). The genome sequence data were uploaded to the Type (Strain) Genome Server (TYGS) available at https://tygs.dsmz.de, for a whole genome-based taxonomic analysis^14,47^. Information on nomenclature, synonymy and associated taxonomic literature was provided by the List of Prokaryotic names with Standing in Nomenclature (LPSN, available at https://lpsn.dsmz.de) ^47^. Determination of the closest type strain genomes was done in two complementary ways: First, MEP_2_18 genome was compared against all type strain genomes available in the TYGS database via the MASH algorithm^48^, and the ten type strains with the smallest MASH distances were chosen. Second, an additional set of ten closely related type strains was determined via the 16S *rDNA* gene sequences. For that, the 16S *rDNA* gene sequence from the MEP_2_18 genome was extracted using RNAmmer^49^ and then BLASTed^50^ against the 16S *rDNA* gene sequences available in the TYGS database. This was used as a proxy to find the best 50 matching type strains (according to the bitscore) for MEP_2_18 genome and to subsequently calculate precise distances using the Genome BLAST Distance Phylogeny approach (GBDP) under the algorithm 'coverage' and distance formula d4^46^. These distances were finally used to determine the 10 closest type strain genomes for MEP_2_18 genome.

For the phylogenomic inference, all pairwise comparisons among the set of genomes were conducted using GBDP and accurate intergenomic distances inferred under the algorithm 'trimming' and distance formula d4^46^. One-hundred distance replicates were calculated. Digital DDH values and confidence intervals were calculated using the recommended settings of the GGDC 3.0^46,47^. The type-based species clustering using a 70% dDDH radius around each of the 14 type strains was done as previously described^14^.

GET_HOMOLOGUES v05052023^30^ and the related package GET_PHYLOMARKERS^15^ were used to compute clusters of orthologous genes from the input genome sequences and to construct the phylogenomic tree. To accomplish this, we conducted a BLAST analysis of the MEP_2_18 genome (CP042864.2) against both the RefSeq and Genome NCBI databases. Subsequently, we identified and selected 34 complete genome sequences of *B. velezensis* and *B. amyloliquefaciens*. Among these, two were sourced from RefSeq (*B. amyloliquefaciens* GKT04 CP072120.1 and *B. velezensis* JS25R CP009679.1), while the remaining 32 genomes were chosen based on BLAST results from the Genome database. We initially tried using *B. subtilis* 168 T to root the tree but eventually used two strains of *B. amyloliquefaciens* for that (DSM7 and LL3). Clusters of single-copy genes were detected by GET_HOMOLOGUES with parameters -M -e. The resulting clusters were subsequently filtered by the GET_PHYLOMARKERS 20,230,108 docker container with parameters -R 1 -t DNA with the aim of identify high-quality, congruent markers whose CDS nucleotide sequences can be concatenated to produce a maximum likelihood molecular tree. Bayes branch support and UFBoot2 bootstrap values of the resulting tree were computed by IQ-TREE^51^. The tree was plotted using FigTree v1.4.4^52^.

### Core genome, pan-genome, singletons, and comparative genomics

Average nucleotide identity (ANI), core genome, pan-genome, singletons, and classification of the cluster of orthologous (COG) and KEGG^11–13^ functional annotation of MEP_2_18 genome was performed through EDGAR pipeline^53^ (https://edgar.computational.bio.uni-giessen.de). ANI was calculated by using the method based on a BLASTN comparison of the genome sequences as described by Goris et al.^16^.

A customized graphical map of the MEP_2_18 genome was constructed using Proksee (https://proksee.ca) ^19^, and several tools from Proksee were used for analysis, such as Comprehensive Antibiotic Resistance Database (CARD) and mobileOG-db.

Global macro-synteny between the closely related strains *B. velezensis* FZB42, TrigoCor1448 and MEP_2_18 were performed using the PanExplorer pipeline^17^.

The web servers ICEfinder^18^, mobileOG-db from Proksee system (https://proksee.ca/), IslandViewer 4^22^, and VRprofile2^23^ (https://tool2-mml.sjtu.edu.cn/VRprofile/VRprofile.php) were used to report the presence of integrative and conjugative elements (ICE), integrative and mobilizable elements (IMEs), genomic islands, and antimicrobial resistance (AMR) annotations, respectively. PHASTEST^20^ and DBSCAN-SWA^21^ were also used to evidence the presence of phage elements.

### Prediction of biosynthetic gene clusters

Sequences for putative Commercial Useful Enzymes (CUEs) were detected through the MetaBioME^24^ web resource (https://metasystems.riken.jp/metabiome/index.php).

The number and types of biosynthetic gene clusters for secondary metabolites (BGCs) in the genome sequence of MEP_2_18 were identified by antiSMASH version 7.0^25^. Complementarily, another unknown and characterized BGCs were identified and genetic similarities in gene clusters were predicted using BAGEL4^26^ and PRISM4^27^ web server tools.

Geneious Prime^®^ 2023.1.1 sequence analysis software was used to identify and compare the fengycin BGC of MEP_2_18 with those from the strain *B. velezensis* FZB42. BGC cluster sequence was downloaded from MIBiG (The Minimum Information about a Biosynthetic Gene cluster) https://mibig.secondarymetabolites.org/.

### Antibacterial activity of CLP and fengycin

The antibacterial activity of CLP and the C16–C17 fengycin fraction produced by MEP_2_18 was tested on LB agar plates against *Acinetobacter baumanii* Ab242, *Streptococcus uberis* strains UB33 and UB56, *Salmonella typhimurium* ATCC 14028, *Proteus mirabilis* and *Escherichia coli*, by using the disk diffusion method as described previously^8^.

Briefly, an aliquot from a culture of each strain (grown until OD_600 nm_ 0.3 to 0.4, the logarithmic phase of growth) was spread on a plate. Different volumes (10 µL for *S. uberis* and *A. baumanii* Ab242, and 20 µL for *Salmonella typhimurium* ATCC 14028, *P. mirabilis* and *E. coli*) of methanolic extract of CLP 50X (concentrated 50 times with respect to the volume of MEP_2_18 culture supernatant used for the extraction of CLP) and fengycin (stock 10 mg/mL) were deposited onto sterile paper disks. The imbibed paper disks were left for 10 min under the sterile airflow to allow methanol evaporation, and then deposited onto the plate. After incubation for 12 h and 6 days (for the Enterobacteriaceae strains) at 37 °C, growth inhibition zones were visualized. Paper discs imbibed with methanol or the acidic precipitate from cell-free supernatant of the JH642 strain were used as controls. The analysis was done in triplicate.

### Supplementary Information

Supplementary Information.

## Data Availability

Data generated in this research are provided as supplementary material with this manuscript. DNA sequence data are deposited in the NCBI GenBank database under the accession number CP042864.2.
