# Supplementary material for: Unraveling the genome of Bacillus velezensis MEP218, a strain producing fengycin homologs with broad antibacterial activity: comprehensive comparative genome analysis
Source: Sci Rep. 2023 Dec 13;13:22168. doi: 10.1038/s41598-023-49194-y (PMC10719345; doi:10.1038/s41598-023-49194-y)
Supplement: Supplementary file 1 — Supplementary Information. [file 41598_2023_49194_MOESM1_ESM.pdf]

Supplementary Information

Unraveling the Genome of *Bacillus velezensis* MEP$_2$18, a strain producing fengycin homologs with broad Antibacterial Activity: Comprehensive Comparative Genome Analysis

Daniela Medeot[1], Analía Sannazzaro[2], María Julia Estrella[2], Gonzalo Torres Tejerizo[3], Bruno Contreras-Moreira[4], Mariano Pistorio[3], Edgardo Jofré[1,*]

[1]Instituto de Biotecnología Ambiental y Salud (INBIAS), CCT-CONICET-Córdoba, Universidad Nacional de Río Cuarto, PC 5800, Córdoba, Argentina.
[2]Instituto Tecnológico de Chascomús (INTECH), Consejo Nacional de Investigaciones Científicas y Técnicas (CONICET) - Universidad Nacional de San Martín (UNSAM), Chascomús, PC 7130, Argentina.
[3]Departamento de Ciencias Biológicas, Facultad de Ciencias Exactas, IBBM (Instituto de Biotecnología y Biología Molecular), CCT-CONICET-La Plata, Universidad Nacional de La Plata, La Plata, PC 1900, Argentina.
[4]Estación Experimental de Aula Dei-CSIC, Zaragoza, PC 50059, Spain

*ejofre@exa.unrc.edu.ar

Supplementary Table 1. COG and KEGG functional classification

Bacillus velezensis strain MEP_218_CP042864

**KEGG**	
Carbohydrate metabolism	196
Energy metabolism	100
Lipid metabolism	69
Nucleotide metabolism	64
Amino acid metabolism	177
Metabolism of other amino acids	45
Glycan biosynthesis and metabolism	69
Metabolism of cofactors and vitamins	151
Metabolism of terpenoids and polyketides	35
Biosynthesis of other secondary metabolites	46
Xenobiotics biodegradation and metabolism	30
Genetic information processing	174
Environmental information processing	256
Cellular processes	139
Organismal systems	35
Human diseases	67
Protein families: metabolism	287
Protein families: genetic information processing	519
Protein families: signaling and cellular processes	528
Unclassified: metabolism	156
Unclassified: genetic information processing	37
Unclassified: signaling and cellular processes	153
Not annotated	1118

**COG**	
RNA processing and modification	1
Chromatin structure and dynamics	0
Energy production and conversion	155
Cell cycle control	197
Amino acid transport and metabolism	265
Nucleotide transport and metabolism	87
Carbohydrate transport and metabolism	246
Carbohydrate transport and metabolism	180
Lipid transport and metabolism	153
Translation	223
Transcription	287
Replication	108
Cell wall/membrane/envelope biogenesis	190
Cell motility	47
Posttrans. Modification	131
Inorganic ion transport and metabolism	160
Sec. metabolites biosynthesis	84

General function prediction only	236
Function unknown	139
Signal transduction mechanisms	199
Intracellular trafficking	33
Defense mechanisms	98
Extracellular structures	4
Transposase	16
Nuclear structure	0
Cytoskeleton	6
Not annotated	429

Supplementary Table 2. Comparison of Genome-to-Genome distances obtained from Type Strain Genome Server (TYGS)

		Type Strain Genome Server						
**Query strain**	**Subject strain**	**dDDH (d0, in %)**	**C.I. (d0, in %)**	**dDDH (d4, in %)**	**C.I. (d4, in %)**	**dDDH (d6, in %)**	**C.I. (d6, in %)**	**G+C content difference (in %)**
MEP218	*Bacillus velezensis* FZB42	97,3	[95.6 - 98.3]	91,6	[89.5 - 93.3]	97,9	[96.8 - 98.6]	0,11
MEP218	*Bacillus velezensis* CBMB205	96,5	[94.7 - 97.8]	84,8	[82.1 - 87.2]	96,7	[95.2 - 97.8]	0,16
MEP218	*Bacillus velezensis* NRRL B-41580	93,3	[90.6 - 95.3]	84,6	[81.9 - 87.0]	94,3	[92.2 - 95.8]	0,27
MEP218	*Bacillus siamensis* KCTC 13613	91	[87.9 - 93.4]	56,7	[53.9 - 59.4]	87	[83.9 - 89.5]	0,25
MEP218	*Bacillus vanillea* XY18	90,4	[87.2 - 92.8]	56,7	[53.9 - 59.4]	86,4	[83.3 - 89.0]	0,27
MEP218	*Bacillus amyloliquefaciens* DSM 7	83,7	[79.9 - 86.9]	55,5	[52.7 - 58.2]	80,5	[77.1 - 83.5]	0,51
MEP218	*Bacillus nakamurai* NRRL B-41091	72,5	[68.5 - 76.1]	30,9	[28.5 - 33.4]	60,5	[57.2 - 63.6]	1,33
MEP218	*Bacillus subtilis* NCIB 3610	32,4	[29.0 - 36.0]	21	[18.7 - 23.4]	28,4	[25.5 - 31.5]	3,2
MEP218	*Bacillus subtilis* ATCC 6051	33,2	[29.8 - 36.8]	20,9	[18.7 - 23.3]	28,9	[26.0 - 32.0]	3,07
MEP218	*Bacillus axarquiensis* NRRL B-41617	33,8	[30.4 - 37.3]	20,7	[18.5 - 23.1]	29,2	[26.3 - 32.3]	2,79
MEP218	*Bacillus rugosus* SPB7	30,3	[26.9 - 33.9]	20,6	[18.4 - 23.1]	26,9	[24.0 - 30.0]	3,47
MEP218	*Bacillus stercoris* D7XPN1	33,5	[30.1 - 37.1]	20,6	[18.4 - 23.0]	29	[26.1 - 32.1]	2,79
MEP218	*Bacillus vallismortis* DV1-F-3	28,6	[25.3 - 32.2]	20,5	[18.3 - 22.9]	25,6	[22.8 - 28.8]	2,83
MEP218	*Bacillus mojavensis* KCTC 3706	33,6	[30.2 - 37.2]	20,4	[18.2 - 22.8]	29	[26.1 - 32.1]	2,92

Supplementary Table 3. List of Genomic Islands obtained from IslandViewer 4

Island start	Island end	Length	Method	Gene name	Gene ID	Locus	Gene start	Gene end	Strand	Product
7015	22177	15162	Predicted by at least one method	URM42879.1	phy_1	D9R10_00050	7015	8166	-1	3-phytase
7015	22177	15162	Predicted by at least one method		capD_1	D9R10_00055	8482	8906	1	dTDP-glucose 4,6-dehydratase
7015	22177	15162	Predicted by at least one method	URM42880.1	yotN_1	D9R10_00060	8940	9116	-1	SPBc2 prophage-derived uncharacterized protein YotN
7015	22177	15162	Predicted by at least one method	URM42881.1	yoqO	D9R10_00065	10920	11294	1	SPBc2 prophage-derived uncharacterized protein YoqO
7015	22177	15162	Predicted by at least one method			D9R10_00070	11514	11904	-1	Uncharacterized protein
7015	22177	15162	Predicted by at least one method	URM42882.1	rapA_1	D9R10_00075	12275	13411	1	Response regulator aspartate phosphatase A
7015	22177	15162	Predicted by at least one method	URM42883.1		D9R10_00080	13401	13583	1	Uncharacterized protein
7015	22177	15162	Predicted by at least one method			D9R10_00085	13892	14909	1	Uncharacterized protein
7015	22177	15162	Predicted by at least one method	URM42884.1	yolB_1	D9R10_00090	14973	15332	-1	SPBc2 prophage-derived uncharacterized protein YolB
7015	22177	15162	Predicted by at least one method	URM42885.1		D9R10_00095	15338	15562	-1	Uncharacterized protein
7015	22177	15162	Predicted by at least one method	URM42886.1		D9R10_00100	15713	16171	-1	SMI1-KNR4 cell-wall
7015	22177	15162	Predicted by at least one method	URM42887.1	yobL_1	D9R10_00105	16174	17979	-1	Ribonuclease YobL
7015	22177	15162	Predicted by at least one method	URM42888.1	ynaB	D9R10_00110	18016	18450	-1	YnaB
7015	22177	15162	Predicted by at least one method	URM42889.1	yokF	D9R10_00115	18715	19680	1	SPBc2 prophage-derived endonuclease YokF
7015	22177	15162	Predicted by at least one method	URM42890.1	yokA	D9R10_00120	19897	21564	1	Resolvase-like protein YokA
7015	22177	15162	Predicted by at least one method	URM42891.1	ypqP	D9R10_00125	21587	22177	1	Polysaccharide biosynthesis protein
711648	723026	11378	Predicted by at least one method	URM43507.1	ytsP	D9R10_03595	711648	712130	1	Protein YtsP
711648	723026	11378	Predicted by at least one method		ytrP	D9R10_03600	712165	713912	-1	Uncharacterized protein YtrP
711648	723026	11378	Predicted by at least one method	URM43508.1		D9R10_03605	713950	714174	-1	Uncharacterized protein
711648	723026	11378	Predicted by at least one method	URM43509.1	rpsD	D9R10_03610	714203	714805	1	30S ribosomal protein S4
711648	723026	11378	Predicted by at least one method	URM43510.1	rapK	D9R10_03615	715098	716210	-1	Response regulator aspartate phosphatase K
711648	723026	11378	Predicted by at least one method	URM43511.1		D9R10_03620	716499	717182	1	Uncharacterized protein

711648	723026	11378	Predicted by at least one method			D9R10_03625	717610	717906	-1	HNH endonuclease
711648	723026	11378	Predicted by at least one method	URM43512.1		D9R10_03630	717973	718719	-1	Uncharacterized protein
711648	723026	11378	Predicted by at least one method	URM43513.1		D9R10_03635	718930	719889	-1	YaaC-like Protein
711648	723026	11378	Predicted by at least one method	URM43514.1		D9R10_03640	719896	720123	-1	Uncharacterized protein
711648	723026	11378	Predicted by at least one method	URM43515.1		D9R10_03645	720398	720580	-1	Uncharacterized protein
711648	723026	11378	Predicted by at least one method	URM43516.1	hmp_1	D9R10_03650	720691	721881	-1	Flavohemoprotein
711648	723026	11378	Predicted by at least one method	URM43517.1		D9R10_03655	722040	722114	1	hypothetical protein
711648	723026	11378	Predicted by at least one method	URM43518.1		D9R10_03660	722120	722347	1	Uncharacterized protein
711648	723026	11378	Predicted by at least one method	URM43519.1	yraA	D9R10_03665	722517	723026	1	Putative cysteine protease YraA
843770	852977	9207	Predicted by at least one method	URM43633.1	hmp_2	D9R10_04390	843770	844945	-1	Flavohemoprotein
843770	852977	9207	Predicted by at least one method	URM43634.1		D9R10_04395	845163	845804	1	Cupin domain-containing protein
843770	852977	9207	Predicted by at least one method		yjcN	D9R10_04400	845865	846185	-1	Uncharacterized protein YjcN
843770	852977	9207	Predicted by at least one method	URM43635.1		D9R10_04405	846507	846743	-1	Uncharacterized protein
843770	852977	9207	Predicted by at least one method		rapH_1	D9R10_04410	847469	848599	-1	Response regulator aspartate phosphatase H
843770	852977	9207	Predicted by at least one method	URM43636.1		D9R10_04415	848695	849093	-1	Uncharacterized protein
843770	852977	9207	Predicted by at least one method	URM43637.1		D9R10_04420	849297	849953	1	Uncharacterized protein
843770	852977	9207	Predicted by at least one method	URM43638.1		D9R10_04425	850041	850214	-1	DNA-binding protein
843770	852977	9207	Predicted by at least one method	URM43639.1		D9R10_04430	850354	851010	1	Uncharacterized protein
843770	852977	9207	Predicted by at least one method	URM43640.1		D9R10_04435	851031	851213	1	hypothetical protein
843770	852977	9207	Predicted by at least one method	URM43641.1		D9R10_04440	851336	851839	1	Uncharacterized protein
843770	852977	9207	Predicted by at least one method		xlyB_1	D9R10_04445	851872	852135	-1	N-acetylmuramoyl-L-alanine amidase XlyB
843770	852977	9207	Predicted by at least one method	URM43642.1		D9R10_04450	852426	852977	-1	Integrase
2334242	2338438	4196	Predicted by at least one method		ywgB	D9R10_12120	2334472	2334974	-1	Putative transcriptional regulator
2334242	2338438	4196	Predicted by at least one method	URM44991.1	azoR1_1	D9R10_12125	2335171	2335812	1	FMN-dependent NADH-azoreductase 1

2334242	2338438	4196	Predicted by at least one method			D9R10_12130	2335853	2336673	1	Nucleoside-diphosphate-sugar epimerase
2334242	2338438	4196	Predicted by at least one method	URM44992.1	azoR_2	D9R10_12135	2336759	2337388	1	FMN-dependent NADH-azoreductase
2334242	2338438	4196	Predicted by at least one method	URM44993.1		D9R10_12140	2337437	2337886	1	Ribosomal protein S18 acetylase RimI
2334242	2338438	4196	Predicted by at least one method		yrkL_1	D9R10_12145	2337966	2338260	1	Uncharacterized NAD(P)H oxidoreductase YrkL
3667794	3686636	18842	Predicted by at least one method	URM46143.1	pps	D9R10_18500	3667794	3670391	1	Putative phosphoenolpyruvate synthase
3667794	3686636	18842	Predicted by at least one method			D9R10_18505	3670667	3671143	1	Uncharacterized protein
3667794	3686636	18842	Predicted by at least one method	URM46144.1		D9R10_18510	3671115	3671213	-1	YjcZ family sporulation protein
3667794	3686636	18842	Predicted by at least one method	URM46145.1		D9R10_18515	3671727	3672149	-1	Uncharacterized protein
3667794	3686636	18842	Predicted by at least one method	URM46146.1		D9R10_18520	3672622	3672807	1	Uncharacterized protein
3667794	3686636	18842	Predicted by at least one method		yisT_2	D9R10_18525	3673205	3673615	1	Uncharacterized protein YisT
3667794	3686636	18842	Predicted by at least one method	URM46147.1	adh	D9R10_18530	3673766	3674782	-1	Alcohol dehydrogenase
3667794	3686636	18842	Predicted by at least one method		yopD	D9R10_18535	3675068	3675403	1	SPBc2 prophage-derived UPF0715 membrane protein YopD
3667794	3686636	18842	Predicted by at least one method	URM46148.1	yoaP	D9R10_18540	3675500	3676261	1	putative N-acetyltransferase YoaP
3667794	3686636	18842	Predicted by at least one method	URM46149.1	ynaD_2	D9R10_18545	3676494	3677036	1	putative N-acetyltransferase YnaD
3667794	3686636	18842	Predicted by at least one method	URM46150.1	yolB_2	D9R10_18550	3677264	3677623	1	SPBc2 prophage-derived uncharacterized protein YolB
3667794	3686636	18842	Predicted by at least one method	URM46151.1		D9R10_18555	3677654	3678088	1	Uncharacterized protein
3667794	3686636	18842	Predicted by at least one method		yoaO	D9R10_18560	3678217	3678695	1	Uncharacterized protein
3667794	3686636	18842	Predicted by at least one method	URM46152.1		D9R10_18565	3678741	3678968	-1	Uncharacterized protein
3667794	3686636	18842	Predicted by at least one method			D9R10_18570	3679252	3679874	1	Uncharacterized protein
3667794	3686636	18842	Predicted by at least one method	URM46153.1		D9R10_18575	3680099	3681172	1	Uncharacterized protein
3667794	3686636	18842	Predicted by at least one method	URM46154.1	gbpA	D9R10_18580	3681380	3682000	1	GlcNAc-binding protein A
3667794	3686636	18842	Predicted by at least one method	URM46155.1		D9R10_18585	3682165	3682263	-1	YjcZ family sporulation protein
3667794	3686636	18842	Predicted by at least one method			D9R10_18590	3682413	3682742	1	Uncharacterized protein
3667794	3686636	18842	Predicted by at least one method	URM46156.1	yvgO_2	D9R10_18595	3683043	3683579	-1	Uncharacterized protein

3667794	3686636	18842	Predicted by at least one method	URM46157.1		D9R10_18600	3683865	3684050	-1	hypothetical protein
3667794	3686636	18842	Predicted by at least one method	URM46158.1	yobO_2	D9R10_18605	3684219	3686636	-1	Putative phage-related protein YobO

Supplementary Table 4. List of mobile genetic elements

Mobile Genetic Elements	Details	Position	Length	GC Content	Antibiotic Resistance Genes	Drug Class	Drugs	Virulence Gene	Virulence Description
Prophage	-	2120338..2156531	36193	42.28	-	-	-	-	-
Prophage	-	665714..690343	24629	48.69	-	-	-	-	-
Prophage	-	1227866..1319677	91811	47.10	-	-	-	-	-
Prophage	-	2916221..2924588	8367	45.74	-	-	-	-	-
Prophage	-	1848671..1859410	10739	42.46	-	-	-	-	-
Prophage	-	1805003..1818816	13813	45.25	-	-	-	-	-
IScluster/Tn	ISBsu1 / ISBsu1	578001..579150	1149	42.47	-	-	-	-	-
IScluster/Tn	ISBsu1 / ISBsu1	687653..688803	1150	42.52	-	-	-	-	-
IScluster/Tn	ISBsu1 / ISBsu1	1805003..1806153	1150	42.43	-	-	-	-	-
IScluster/Tn	ISBsu1 / ISBsu1	2212401..2213539	1138	42.62	-	-	-	-	-
IScluster/Tn	ISBsu1 / ISBsu1	2916221..2917371	1150	42.52	-	-	-	-	-

Supplementary Table 5. List of CARD Resistance Gene Identifier

RGI Criteria	ARO Term	Detection Criteria	AMR Gene Family	Drug Class	Resistance Mechanism	% Identity of Matching Region	% Length of Reference Sequence
Strict	clbA	protein homolog model	Cfr 23S ribosomal RNA methyltransferase	lincosamide antibiotic, streptogramin antibiotic, streptogramin A antibiotic, oxazolidinone antibiotic, phenicol antibiotic, pleuromutilin antibiotic	antibiotic target alteration	99.71	100.00
Strict	FosBx1	protein homolog model	fosfomycin thiol transferase	phosphonic acid antibiotic	antibiotic inactivation	62.77	106.52
Strict	BcI	protein homolog model	class A Bacillus cereus Bc beta-lactamase	cephalosporin, penem	antibiotic inactivation	62.42	99.35
Strict	qacJ	protein homolog model	small multidrug resistance (SMR) antibiotic efflux pump	disinfecting agents and antiseptics	antibiotic efflux	44.9	109.35
Strict	qacG	protein homolog model	small multidrug resistance (SMR) antibiotic efflux pump	disinfecting agents and antiseptics	antibiotic efflux	42.45	113.08
Strict	qacJ	protein homolog model	small multidrug resistance (SMR) antibiotic efflux pump	disinfecting agents and antiseptics	antibiotic efflux	37.37	97.20
Strict	vanY gene in vanB cluster	protein homolog model	vanY, glycopeptide resistance gene cluster	glycopeptide antibiotic	antibiotic target alteration	34.87	101.49
Strict	vanT gene in vanG cluster	protein homolog model	glycopeptide resistance gene cluster, vanT	glycopeptide antibiotic	antibiotic target alteration	34.52	54.63
Strict	vanT gene in vanG cluster	protein homolog model	glycopeptide resistance gene cluster, vanT	glycopeptide antibiotic	antibiotic target alteration	33.69	55.20
